# Challenges, Barriers and Strategies to Improve the Community’s Participation in the Management of Quality and the Accreditation in Chile’s Primary Healthcare Centers

**DOI:** 10.3390/healthcare14203556

**Published:** 2026-10-21

**Authors:** Juan Coss-Mandiola, Jairo Vanegas-López, Alejandra Rojas, Constanza Olivares-Silva, Raúl Carrasco

**Affiliations:** 1 Facultad de Ciencias Médicas, Escuela de Obstetricia y Puericultura, Universidad de Santiago de Chile, Santiago 8320096, Chile; jairo.vanegas.l@usach.cl (J.V.-L.); constanza.olivares@usach.cl (C.O.-S.); 2 Carrera de Obstetricia y Puericultura, Facultad de Medicina y Ciencias de la Salud, Universidad Central de Chile, La Serena 1710164, Chile; alejandra.rojas@ucentral.cl; 3 Departamento de Contabilidad y Gestión Financiera, Facultad de Administración y Economía, Universidad Tecnológica Metropolitana, Santiago 7500998, Chile; rcarrascoa@utem.cl

**Keywords:** community participation, primary health care, quality management in healthcare, health accreditation

## Abstract

Objective: To analyze the level of community participation in quality management and accreditation processes in accredited Family Health Centers (FHCs) in Chile. Methodology: Analytical qualitative study based on the socio-critical paradigm and Participatory Action Research (PAR), reported under the Consolidated criteria for Reporting Qualitative research (COREQ) guidelines. Through purposive maximum variation sampling, 106 key stakeholders (70 healthcare professionals/managers and 36 community leaders) were recruited from eight accredited FHCs. Data collection included eight focus groups structured using the Logical Framework methodology. Data were processed using hybrid (deductive–inductive) qualitative content analysis supported by researcher triangulation. Results: Community participation was shown to be predominantly ritualized and symbolic, confined to passive or informative roles, and sustained mainly by older adult leaders. The main structural barriers include: the invisibility of the community dimension in technical accreditation guidelines, the hegemony of the biomedical–bureaucratic model, clinical workload, scarcity of resources, technical hermeticism, schedule incompatibility, the primacy of quantitative healthcare performance targets, marked knowledge and information asymmetries, and organizational barriers that exclude younger populations. This results in a missed opportunity to incorporate the community’s perspective into continuous process improvement, as well as a decline in the community’s sense of belonging and involvement, leading to purely formal accreditations and the weakening of social control. Proposed strategies prioritize critical health literacy, co-designed participatory monitoring, and the territorialization of local governance. Conclusions: There is a marked dissonance between Primary Healthcare (PHC) public policy and institutional practice; thus, community participation in quality management and health accreditation at this care level remains incipient and symbolic, relegated by systemic barriers to reactive responses. Ensuring legitimate accreditation requires leveraging local commitment and moving from consultative participation toward a model of substantive, deliberative, and oversight co-management with the community that guarantees genuinely binding social control.

## 1. Introduction

The Alma-Ata declaration establishes the right and duty of the population to participate, individual and collectively, in the planning and execution of their health, fomenting self-development and the creation of community skills [1]. In public health, Participation by the Community (PC) is defined as a relationship of dialogue and dynamics between citizens, professionals, and authorities to influence in decision making [2]. Under this framework, various countries have adopted multiple methodological focuses to fulfill World Health Organization (WHO) mandates in regard to PHC [3]. Nowadays, PC is a fundamental pillar of quality management and sanitary accreditation, thus, its implementation is key to empower communities, optimize local resources, ensure accountability from the suppliers, and improve end user experience in the healthcare system [4].

Following this line of argument, and according to the WHO’s own assessments on these topics, a Colombian study is worth highlighting, in which a group of PHC nurses, through a community participation and entrepreneurship program, managed to empower local Afro-Colombian female leaders of Guapi, on individual, organizational, and community levels, achieving greater spirituality, cultural competences, and growth in team decision making in conjunction with government and private institutions [5].

Another strategy worth highlighting that can serve as a good example of how to strengthen PC is proposed and developed for the Andalucia council in Spain, the “School of patients”, which has the objective of improving health and quality of life of those suffering from disease, particularly chronic illnesses. To this end, the school facilitates the exchange of knowledge acquired by living with the ailment. With this, patients, family members, and caretakers can also share their experience with other people, with the healthcare system itself, and society in general [6].

Despite all, there is a lack of coherence in the methods employed by PC, generating uncertainty and barriers for its implementation—for example, the lack of institutionalization in PC [7]. In Chile, PC in healthcare remains mostly on a level of appeasement, not able to reach the degree of citizen power described by Arnstein [8]—that is, not able to achieve real citizen power [9]. With this in consideration, PHC management requires clear tools of evaluation and accreditation that ensure quality and continuous organizational improvement [10,11].

On an international scale, systematic PC interventions were evaluated to be potentially less costly and could be utilized to complement already existing strategies to improve quality of life, predominantly techniques with minimal end user and community involvement [12]. On the other hand, a study realized in China using semi-structured interviews managed to generate a clear profile of high-performance PHC organizations from the patient’s perspective. Through this perspective, it was possible to identify that patients expected a PHC organization that provided integral healthcare services through an integrated focus; a PHC organization close to home, with a clean and orderly environment and managed by the government [13].

Since 2001, Chile has employed the Model of Comprehensive Family and Community Health Care (MCFCHC), fostering collaborative social participation between the community and institutions to solve local challenges [14]. This model seeks a transition from paternalism to a deliberative participation with real incidence [15]. To this end, strategies like Local Development Councils (LDC), participative budgets, and citizen’s polls are employed, all being mechanisms that allow the population to directly influence the design and decisions made in healthcare policy [16].

This normative framework links healthcare quality and PC to ensure an integral service that satisfies the user’s necessities [17]. At the same time, it promotes evaluating efficiency and involving the community in the gauging results, accessibility, and satisfaction within the continuous improvement processes [14]. Law No. 19937 [18] regulates the quality assurance in Chile, demanding minimum safety standards, technology, and competencies for service providers both public and private. FHCs, classified as open, low-complexity service providers are evaluated under dimensions that include the patient’s dignity, clinical management, safety of the installations, and record keeping among others [19]. A critical point is the normative update of Decree 29 [20] of 2025, establishing an ambitious, concrete goal, demanding that by the first of July of 2028, 80% of FHCs in the country must be mandatorily accredited. This framework seeks to standardize assistance practices, guaranteeing that PHC is up to valid levels of safety and organizational efficiency [21].

Regarding PC in the field of quality management and accreditation, it is internationally recognized that the capability of healthcare systems to improve the quality of care and control costs require effective cooperation that relies on informed, involved patients [22]. There is also evidence pointing towards political stakeholders increasingly believing that patients can take different roles in medical care to both cut costs and improve quality [23]. On a separate item, guaranteeing participation of the community and the people’s protagonism in the primary healthcare process is currently recognized as a key component in the planning and development of healthcare services [24].

Nonetheless, in Chile a public healthcare issue can be observed, where the community still maintains a receptive role in quality and accreditation. Despite the slow recognition of PC in healthcare from both users and service providers [25], it is fundamental for the accreditation of FHCs to require the involvement of all those who partake in the process (7). While regulations limit PC to standards that “Respect the patient’s dignity” and “Safety in the installations” [26], this coincides with low involvement levels reported by healthcare teams, who see user participation as an aspect to be strengthened, surpassing current experiences which are limited to simulations [27].

The present manuscript reports specifically the first-stage diagnosis phase in a triennial project: Phases of action planning, implementation, and evaluation of the action–reflection cycle are later stages of the project and not object of this report. The study seeks to strengthen PC, a term used in the entirety of the reporting document to refer unequivocally to the intervention of the community involved in the processes of quality management and PHC accreditation in Chile. The utilized design favored spaces of horizontal dialogue between the community assigned to each Family Health Center (FHC) and the healthcare workers with the purpose of highlighting and reinforce the existing participation mechanisms.

In virtue of this, the central purpose of this study was to analyze the levels of PC in healthcare regarding topics related to quality management and accreditation of already-accredited FHCs, taking into consideration the analysis of barriers, effects, and proposed improvements for the PC process in the mentioned topics. Furthermore, an indirect objective of this research has been to contribute information and knowledge regarding the benefits of PC in the subjects of quality and accreditation of healthcare in APS. And as previously stated, this diagnostic phase is part of a triennial project and included leadership training and ontological coaching with the goal of strengthening technical and conversational competencies in local teams and their communities to further support working with the community in these critical areas of public health.

## 2. Materials and Methods

### 2.1. Study Design

The design corresponds to a qualitative study framed in the PAR investigation methodology, following the classic cycle of diagnostic, planning, action–reflection within the PAR framework, and employing Kemmis’s focus [28]. All of this is framed within the socio-critical paradigm that seeks for every participant to question the structures, routines, and arguments that limit its practice, freeing them from institutional or systematic impositions. The theory is built on trying ideas directly in the performance (ideas in action), making the investigation immediately useful for those who carry it out and considering that true PAR only exists when the group cooperates in the transformation of its own practices. There is no separation between the expert researchers and the subject researched [29].

Consistent with the PAR focus, the design has explicitly incorporated the following participative elements:

(a) Participant’s role: Healthcare workers and members of the community were not merely informed but also co-authored the diagnostic and action proposals.
(b) Decision making: The priority topics were jointly defined between the research team and the Health Officials (HO) and Community Members (CM).
(c) Reflection–Action cycle covered in this report: Participative diagnostic allowed the joint identification of barriers and the collaborative construction of solution proposals (not including yet either implementation or results evaluation).

The study is reported following Consolidated criteria for Reporting Qualitative research (COREQ). Consisting of a 32-item verification list with three domains: (i) research team and reflexiveness, (ii) study design, and (iii) analysis and data reporting. This helps researchers inform about important aspects of the research team, study methods, study context, findings, analysis, and interpretation [30].

### 2.2. Context and Participants

The study included HO and CM from 8 accredited FHCs, located in the municipalities of Copiapó (Atacama region) and Chillán (Nuble Region), making use of a prior four-year collaboration between the research team and the aforementioned centers, which facilitated the project’s coordination [27]. Another argument for this intentional sample selection was that it favors the purpose of this study, which seeks the strengthening of PC in quality management and accreditation instances. On this note, the FHCs selected are municipal establishments which have an approximate coverage of between 65% and 70% of the municipal population [31].

### 2.3. Sampling

Intentional. It was planned to incorporate between 10 and 20 HO and between 6 and 20 CM with roles of community leaders in LDC for each FHC. Recruitment was conducted through formal invitation addressed to HO and CM to participate in work meetings related to the project.

### 2.4. Effective Participation and Attendance Rate

In total, 106 people participated (70 HO and 36 CM), with relevant variations between centres (between 7 and 20 participants per FHC, further detailed in the Results section). Regarding effective participation of HO and CM, it was planned to count with a minimum of 80 HO, 70 being those available to work with, whereas it was planned for a minimum of 48 CM to partake, but only 36 of them attended. The main motive behind the low HO attendance was the lack of scheduled hours for management or otherwise non-clinical labor. And in the CMs case, lack of time due to either an overlap with work hours or the existence of other commitments derived from their position as community leaders were identified among the main drivers behind the low attendance.

### 2.5. Recognized Limitation to Participation

It is worth noting that, even though it was planned to count with a higher number of CM, the PC was composed in its majority by LDC leadership, particularly the senior citizens who are in their majority pensioners. It is accepted that this composition is a product of channeling the invitation through municipal health authorities and existing leadership structures, which could have induced a selection bias to individuals with an already higher prior institutional involvement, under representing the youth, those of working age, and other community groups such as uninvolved migrants. This limitation is further mentioned in the Limitations section.

### 2.6. Procedure

This first stage of the project has been structured in two jointly developed phases by the research team, the healthcare teams, and the community.

Phase 1: Consisted of coordinating labors with the municipal healthcare authorities to contextualize the problem, socialize the project, and define jointly with each FHC participant. A presential meeting was held with the municipal healthcare direction teams of both municipalities.

Phase 2: Consisted of the participative diagnostic through which the dialogue between HO and CM was facilitated through group discussion sessions (see data gathering Section 2.7), utilizing the logic frame methodology [26] to jointly structure the problem, causes, consequences, and preliminary solution proposals.

All the results derived from this phase (diagnostic), including all action proposals, were validated simultaneously by the participants through discussions held during the making of each focal group. After this, all the gathered data was systematically logged and sent to each FHC for its review and final validation. This phase included feedback for each FHC. Later, in a second visit, a new meeting and group discussion will be held to consolidate metric validation for each proposed action.

Additionally, the first part of a refining “Leadership, people and team management from an Ontological Coaching perspective” course aimed at HO and CM has already taken place. It is worth highlighting that this formative activity was reported merely as contextual information for the project, being a complement to the diagnostic phase and not part of the qualitative analysis of this manuscript. For that reason, its eventual impact in PC will be a line of analysis left for the project’s later stages.

### 2.7. Data Gathering

The information was gathered through focus group sessions conducted in each FHC by two members of the research team and the joint participation of HO and CM. Each session used a semi-structured discussion guideline elaborated from prior literature and steered by the team. Its validation was based on prior studies made by the same investigative team [32], which allowed the construction of questions geared towards reaching consensus regarding the problem, identify barriers, consequences, and proposed actions to improve PC in quality management.

Eight focus group meetings were held in total, one for each FHC; each meeting had an approximate duration of 3 to 4 h, and they were moderated by two members of the research team, all with a master’s degree formation and experience in prior qualitative research. The strategies to approach power asymmetries present due to HO and CM were sharing a discussion space and the neutral presence of facilitators (researchers) to reduce the risk of institutional hierarchies inhibiting the expression of critical opinions by the community.

Data saturation and/or informative power was defined in function of the coverage criteria of the eight participating FHC. In this case, with each meeting moderated by members of the research team and with a semi structured guideline, data gathering would close each time answers were not found or started to repeat.

To clarify, the data saturation in focal groups was determined when the discussion of a given subject started to reiterate itself. In these cases, it coincided with previously expressed positions without adding new elements, or in a final instance, when the group interaction entered a state of consensus, polarization, or discursive exhaustion. In these cases, the mediator team would intervene by asking questions that allowed the group to either dislodge or close the subject.

### 2.8. Presentation and Results Analysis

The sessions were audio recorded and rigorously transcribed and anonymized through a code that allowed association of the contents with each HO or CM group of each FHC. Example: HO-C1; CM-C1 (Healthcare Worker FHC 1; Member of the Community FHC 1). Additionally, field notes were registered during each session, which were contrasted and verified with the participant groups.

Content analysis was organized following the logic framework (problem, causes, consequences, and preliminary solution proposals), complemented with a qualitative codification process. Qualitative analysis of the transcripts was carried out by two researchers in an independent manner, employing a mixed codification focus. Starting from a deductive structure based on a preliminary concept codebook, which was iteratively refined through inductive analysis to integrate topics and sub-categories emerging directly from the data. To guarantee trustworthiness and methodological credibility, both team members analyzed autonomously the totality of transcriptions; discrepancies and inconsistencies in code assignment were solved through periodic consensus meetings between both researchers. For those cases in which discrepancies persisted, a third researcher intervened to reach a definitive codification by consensus.

### 2.9. Methodological Rigor

From a qualitative methodology perspective and considering as a base the classic rigor criteria [33], the design of this PAR-centric study and the use of the logic framework in eight accredited FHCs in Copiapó and Chillan considered the application of diverse methodologic strategies, with the objective of strengthening trustworthiness and rigor in the analytic process [34].

Credibility was safeguarded through the validation of findings with the participants (member checking) during the meetings of return and consensus of results. Reliability was secured through codification and joint analysis from a multidisciplinary research team, guaranteeing internal consistency through an audit process based on the anonymized transcriptions of audios and the logic framework structure.

Likewise, confirmability was guaranteed through the rigorous keeping of field notes, analytic memos, and direct tracing between emerging discourse and problem trees built, preserving neutrality in the analysis. Transferability of the findings is sustained in the dense context description of the eight accredited FHCs of Copiapó and Chillán and the characterization of the 106 participants, having to exercise caution in this interpretation with respect to non-accredited centers or other regions. Finally, multiple triangulations combining data sources (cross perspectives between HO and CM), gathering techniques (group logic framework guidelines and field notes), and researchers (interdisciplinary review of the study team) all provided a greater depth and consistency in the conclusions.

### 2.10. Ethical Declaration

It is worth mentioning that this study counts with the proper authorization of the Institutional Ethics Committee of the University of Santiago de Chile, which analyzed and authorized all the documents used in this investigation, including informed consent, guidelines for group and individual interviews, and commitment letters from each FHC. All this information is contained in the Ethical Report N°: 250/2025, Santiago, 14 April 2025.

## 3. Results

### 3.1. Defining Study Group

This study included a total of eight FHCs from two regions (Atacama and Ñuble) and two municipalities (Copiapó and Chillán). In the discussion activities (diagnostic), 106 people participated in total between each FHC’s HO and CM. Regarding the main traits of the participants, it was found that HO and CM ascribed to each FHC were 71.7% (76) female and the remaining 28.3% (30) were male. A total of 66% (70) of the participants were HO and 34% (30) were CM. The FHC Candelaria de Rosario in Copiapó had the highest individual participation with 20 participants. Opposite to this, FHC Ultraestación in Chillán had the lowest, with just seven participants (Table 1).

Regarding CM participation, it fluctuated between 1 to 10 participants for each FHC with an average of 5. Regarding CM ages, they fluctuated between 49 and 84 years with an average of 67.9 years. It is worth noting that 69.4% (25) were senior citizens (65 years or older). Gender-wise, 75% (27) were female and the remaining 25% (9) were male. Regarding profession or trade the CM practice, a majority were already retired and/or pensioners, some possessed higher education and many were exclusively dedicated to leadership role. The average amount of time since they joined the organization was 12.4 years. It is also worth highlighting the CM participation observed was lower than planned and expected in each FHC. Some of the reasons for this low participation are described in the discussion section.

**Figure 1:**
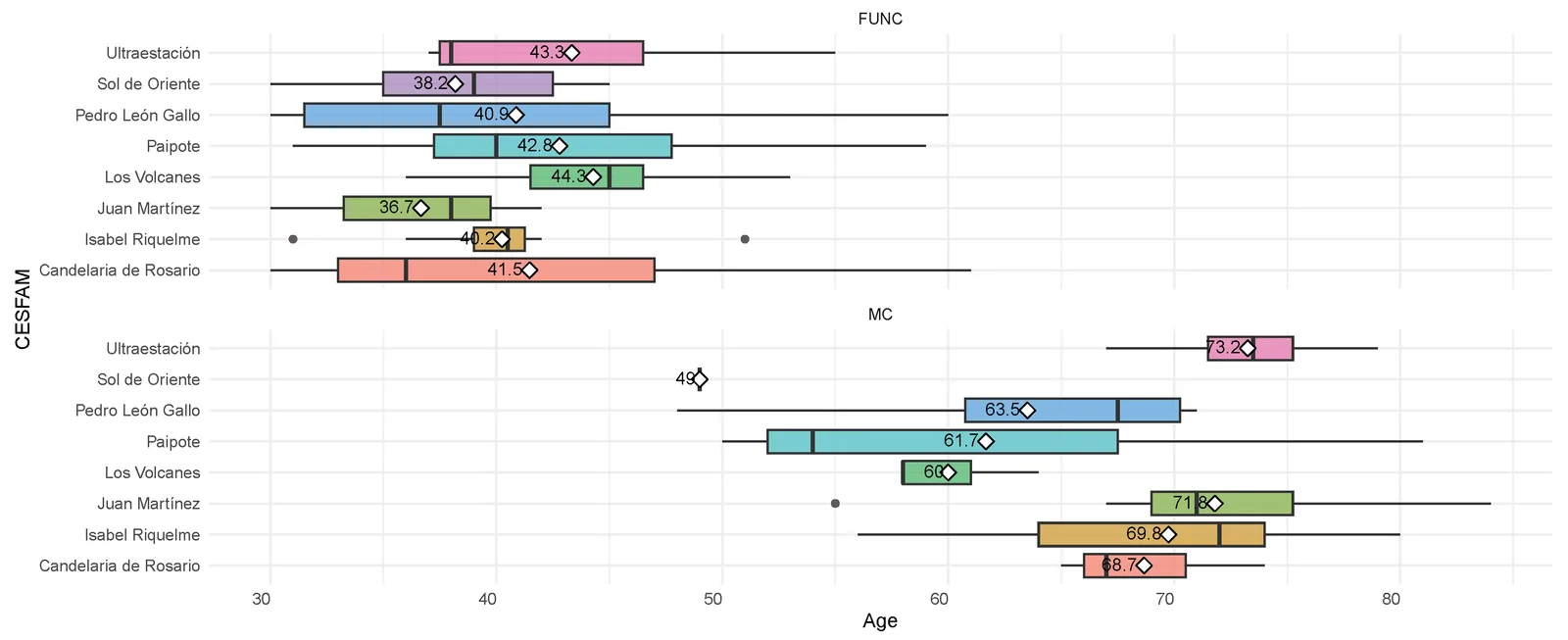
Age distribution of HO and CM by FHC.

Figure 1 shows the age distribution of HOs and CMs, where the age difference between HO and CM can be seen.

**Figure 2:**
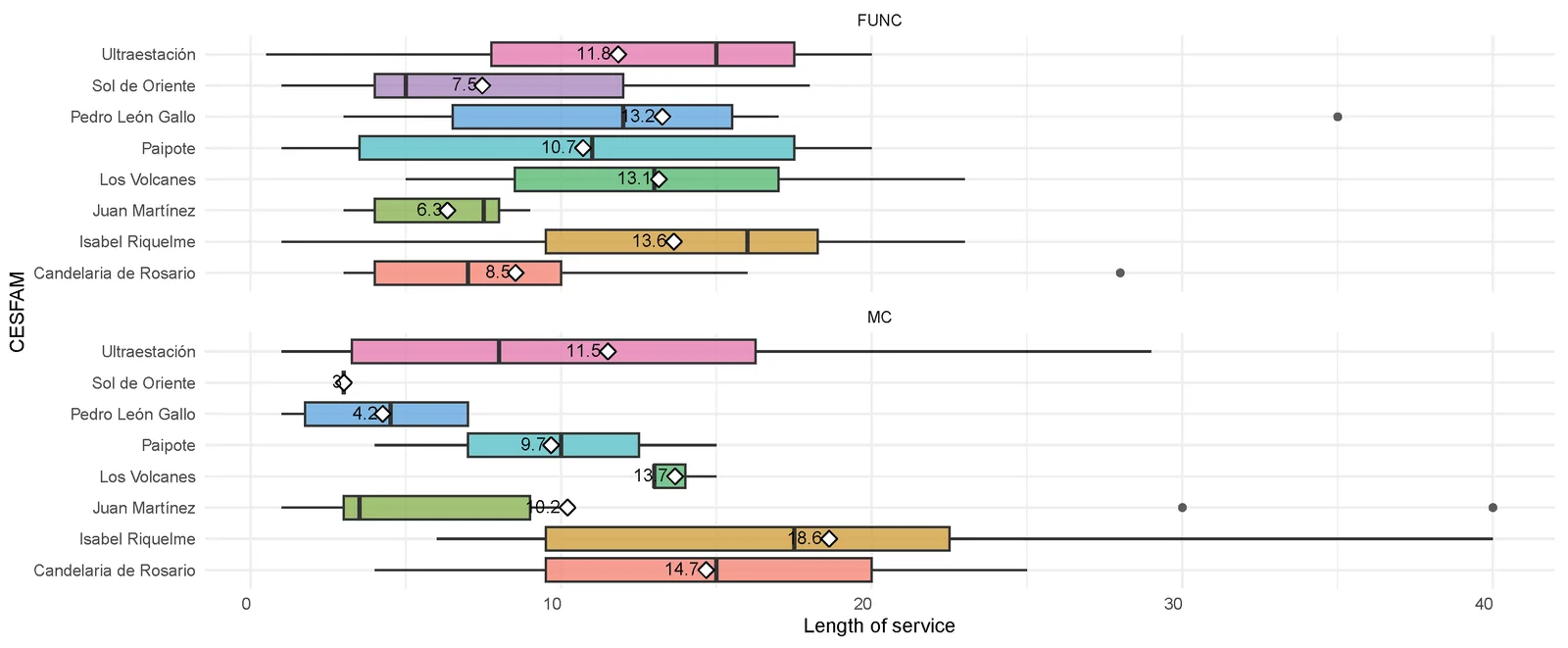
Distribution of length of service for HOs and CMs by FHC.

Figure 2 shows the length of service of HOs and the length of time CMs have been working at the FHC.

Moving on to the HOs, 70% (49) of the participants were female and the remaining 30% (21) male. Regarding their trades and professions, there is a notable presence of obstetricians, nurses, Medics, odontologists, kinesiologists, higher-level nurse technician, administrative personnel, social workers, and computer engineers among others. The results also reflect a low participation of both higher-level nurse technicians and administrative personnel, which participated in a low percentage in the majority of FHCs. The average time since they started working at their FHCs was 10.2 years.

Regarding participation in the “Leadership and management of people and teams from an Ontological Coaching perspective”, 56 individuals attended, a majority of them HO from those same FHCs. Regarding the impact evaluation of the learning derived from the course, they will not be treated in this manuscript.

### 3.2. Relevant Results Related to the PC

Qualitative inductive-deductive analysis of the narratives presented by participants (CM and HO) from the eight studied FHCs allowed a reconstruction of the socio-phenomenological structure of the PC in PHC and its articulation with the quality management and accreditation of healthcare. The findings are organized in four nodal, interconnected categories: (1) diagnostic and perception of PC in the primary ambit, quality and accreditation; (2) causes and determinant barriers for low participation; (3) effects and socio-institutional implications of the attention model and social control; and (4) action course and strategic proposals for community reconfiguration.

For the detailed analysis, in vivo codes were included (a qualitative codification technique in which an exact word or brief phrase expressed by the participant is used as a tag or name of a conceptual category)—for example, taking the exact phrase (Participation is scarce) to denominate the category dealing with PC perception. Its function is to name and classify data.

#### Categoric Matrix of Qualitative Findings

Table 2 provides a summary of emerging topic architecture, articulating analytic categories, sub-categories, in vivo codes, and the interpretative lectures.

1. Perception of PC in primary care and quality management

**1.1. General Diagnose: Limited reach, worn leadership, and “Rally bias”** Broad discourse analysis reveals an extended consensus between HOs and CMs regarding a low level of citizen involvement in healthcare and promotion activities in the eight studied FHCs. Qualitative participation is not only notorious for its numeric scarcity but also for a recurrent pattern of low territorial representativity. Institutional discourse evidences a systematic clash when the rallying efforts meet an insufficient and lukewarm community response.

*“I started assisting to the development council last year. Sometimes attendance is low, right? It is low, isn’t it? It is, and it has been attempted to integrate the community bit by bit, but nonetheless the whole participation issue, all being the same, it remains low.”* (HO-C1).

This scarcity is analytically translated in what key actors define as a *“Rally bias”*, determined by repetitive concentration of community work in a reduced leadership elite. The lack of generational turnover causes involuntary co-optation of dialogue spaces by the same historic representatives which make up the plurality of voices in local planning.

*“Not only topics, it’s in the healthcare sector. It is hard to rally folk for community work, always the same leaders that work with us, for example, same leaders for quality processes, we told them as it truly is…but attendance is always low, even in educational health related activities”* (HO-C5).

From a community perspective, the decline in participation can be viewed through two complementary lenses. First, partial satisfaction of immediate care necessities induces a sort of accommodation or withdrawal from the citizens.

*“Our council, let me make a bit of history. Today is not what it once was. Before people participated a whole, whole lot. Right? Very participative. I believe that, perhaps, it’s now that all their needs are solved, maybe nowadays they don’t want that participation”* (CM-C5).

On a different note, the leadership perceives a methodological obsolescence in institutional rally mechanism. Participation tends to be formal and rigid, creating a rift between the FHC’s agenda and the day-to-day territorial reality.

*“The community’s participation in meetings is really limited, really partial, few rally and very few participate. We have to change the methodology, here Mr. HO is changing it because he’s now going to the neighbors association, to the elderly clubs”* (CM-C7).

This methodological obsolescence is deepened by the emergence of a phenomena of “resistance to participation”, founded by mistrust towards the healthcare system due to the persistent rift between the adopted commitments by the authorities in community instances and the actual tangible outcomes.

*“There’s this really complex phenomenon going on now, which is resistance to participation. How so? Each time less and less people want to participate in activities, meetings, no matter what it is. Maybe the authorities have done a lot of overpromising and underdelivering. They organize meetings…they always talk the same, they seek participation but at the end, let’s say, the results are too few”* (HO-C7).

**1.2. Community participation in accreditation processes: From “Passive entity” to the perceived rift.** When examining specifically the community’s involvement in the accreditation cycles of healthcare quality, the qualitative narrative manifests a structural disassociation. PC in this ambit has been historically tangential or practically non-existent, configurating a scenario notorious for the lack of representation in operative meetings and citizens’ inactivity when it comes to technical decisions.

*“Well, truth is, I see little participation from the community. Ok. Because there’s like three representatives here and that’s it from the whole community”* (CM-C2).

*“Truth being, I think that in the three processes of quality accreditation we already have and now the fourth one incoming, I feel the community hasn’t been so active.”* (HO-C2).

Even though an institutional perspective recognizes the value that integrating the user’s perspective could bring to enrich and validate care improvement projects: *“Ideally more people in the community would get involved as to contribute with ideas…”* (HO-C2), an explicative tension between the community’s self-responsibility: *“There too is a Mea Culpa from the community for not wanting to participate.”* (HO-C2), and the biomedical bias originating from the accreditation design itself.

A critical finding resides in the historic assumption by health teams that quality accreditation constitutes a subject of eminently clinically standardized domain. Under this implicit premise, the user was rendered invisible, conceived as a merely inert receptor of care.

*“…when we first got accredited…the standardization of procedures we wanted to see o present were mainly and eminently clinical. So, I think that maybe it was our mistake…we didn’t think it through. We never thought that it was the community because we thought that, even though they are recipients of these processes…we didn’t think it necessary. Because we kind of assumed that they had nothing to do with this, rather, they were going to be a passive entity that were going to receive the product”* (HO-C3).

The key moment that interrupted this institutional inertia is linked to moments of celebration or the formal end of accreditation processes. Ritual instances in which community rapport emerges, triggering a sort of conscience or *“critical awakening”* in healthcare personnel.

*“Now, me personally when it was like the lightbulb went a bit on with the issue about community presence in accreditation processes was when the ceremony to celebrate the FHC # got accredited took place…a community member showed up and was the president of the Development Council saying: ‘We are all here together celebration something, something we weren’t even included to work on’”* (HO-C3).

This public questioning allowed raising the problems with accreditation and transformed the exclusion into an explicit *“felt rift”*, both for the directive teams and the organized community:

*“And that was what kind of opened the path a bit for us to also see that it was a rift, a rift felt by the community and that we had to bridge it somehow”* (HO-C3).

*“Truthfully, I work with the community. I feel like we lack a lot. And that was one of the largest rifts that maybe got evidenced…the instructions sent from the ministry…wasn’t oriented towards the community.”* (HO-C4).

Regardless of this progressive recognition, the alienation remains in social leadership regarding the technical labor of accreditation. As it was conceived as a merely administrative or patrimonial requirement, the user assumes a distant, passive outlook.

*“The community doesn’t intervene in accreditation; the community knows that it was or wasn’t accredited but doesn’t intervene. I think it belongs to the people that work there, the director and all the staff. Us as community leaders have nothing to do. If they ask our opinion, yes, but if they don’t ask we don’t tell.”* (CM-C7).

In summary, when observing the specific results derived from the dialogue process with HOs and CMs, the perception of low PC was identified as an issue both generally and specifically in topics regarding quality and healthcare accreditation.

In respect thereof, both in HOs and CMs, it is observed that PC is perceived as a diminished phenomenon, both in formal and informal settings such as LDC. Participants mention different causes for this low PC. On one hand, a resistance to community participation has been identified, with a difficulty in rallying the community towards active participation. In the other, this meager participation is observed as a very partial informative instance.

Consequently, results suggest that the issue does not involve a total lack of participation, but a lack of depth, reach and incidence. While incipient experiences have been identified, involving participation and specific topics regarding patient’s dignity in which the users can perform an active role regarding quality, levels of participation are still predominantly initial. Accreditation, in this scenario, risks remaining an institutionally significant process, but with a limited social appropriation. The challenge thus consists in transit from participation centered in information, queries, and grievances towards substantive participation forms that allow the incorporation of community experience in the definition of issues, prioritization of necessities, decision making, and quality evaluation.

2. Determining factors and fundamental barriers to PC (causes)

**2.1. Institutional dimension and normative. Invisibility in accreditation guidelines and biomedical hegemony.** One of the most important explicative findings is related to the structural omission of community participation in the normative frameworks for quality accreditation in Chile. Testimony from those in charge of quality and directive teams emphasize unanimously that the standard general accreditation guidelines for open care lacks mandatory traits or indicators that demand the effective inclusion of citizens.

*“The quality guideline doesn’t allow or does not ask for community participation, so I think that’s one of the things…Here I have three people in charge with quality assurance that have been here and they ask for nothing, nothing that says the community has to do this to fulfil this trait.”* (HO-C8).

“*The directions from the ministry were not geared towards the community”* (HO-C5).

*“What happens is that the quality guidelines do not ask for community participation, there should be some traits that directly integrate the community.”* (HO-C8).

These normative omissions validates and perpetuates endogamic institutional practices, where the quality evaluation is restricted to technical committees constituted exclusively by healthcare workers, squandering the expert wisdom of the community.

*“One of the great flaws nowadays is that these (quality) meetings have not been generated with the community…There’s much contribution the community ought to give us and nowadays we are not making it jointly”* (HO-C4).

To this normative bias, we can add the persistence of a clinical-service-centric hegemony in the FHCs’ organizational culture. The promotion of health as a mandate and the networking ends up displaced by the urgency of individual healthcare (*“We need more community work…we focus on clinical”* HO-C1), consolidating a reactive care model.

**2.2. Financialization of health, performance goals, and reduced resources.** In a structural plane, arguments place the pressure for production performance and achieving budget goals (sanitary goals and Primary Health Care Activity Indicators (PHCAI)) as decisive barriers that asphyxiate participation. The relentless demand to fulfill hourly performances by professionals effectively eliminates the “protected time” needed to articulate extracurricular community activities.

*“It’s hard to reach the community, close the schedule because there’s this issue with performance that is demanded and I know the disposition of the workers is there, it’s not that we don’t want to.”* (HO-C1).

*“The schedule is demanded from the workers according to performance, which sometimes is not enough to deliver a sufficient and usable health education, especially for seniors”* (HO-C7).

*“We do not have protected time to communicated…we lack protected time for community work”* (HO-C7/HO-C3).

This dynamic produces a paradoxical tension: while centralized evaluation systems reward volume of clinical production, the community punishes the lack of available hours, defining quality not by accredited processes but the opportunity of access to care. (*“If they do not have time, people say it’s bad quality”* CM-C4).

Likewise, primary evidence shows a progressive reduction in financing for promotion and prevention, both areas having their funding decimated to favor closed clinical programs. This reduces participation to a diminished, precarious, and symbolic expression.

*“…there used to be more emphasis on promotion and prevention, each time there have been less resources in that regard…nonetheless they’re always asking or increasing all our goals and sadly that’s number. We’re seen as numbers alright? And if we do not keep up with those numbers there’s no money.”* (HO-C2/HO-C7).

*“For participation and promotion, we have to make beckoning things. Have coffee, buy the cups and all that comes from our resources.”* (HO-C1).

*“Fake participation if there is no budget.”* (CM-C6).

This financial scarcity is also joined by severe physical infrastructure and exclusive personnel limitations for managing community networks.

*“There isn’t any time resource…we do not have the physical space to tell them; we invite them to our house”* (HO-C5).

*“There are a lot of sub-programs which diminish our scarce resources”* (HO-C1).

**2.3. Communication dimension and asymmetry in technical-sanitary management.** The third structural cause shows a relationship with both information asymmetry and the high complexity of the technical lexicon in which the quality standards are framed. Though institutions report having shared the accreditation goals in institutional memos and social media, the information does not manage to transform into significant knowledge for the citizens.

*“Even though the community has received the information as to what is accreditation…people retain little of that. They really do not manage to understand it deep down what its accreditations truly means and that makes them not to differentiate or appreciate between an accredited FHC and one that is not”* (HO-C4).

*“The information doesn’t reach us neighbors”* (CM-C3).

This informational gap is further aggravated by a bottleneck in traditional transmission channels: the information given by the health team is transferred in a concentrated manner to few development council leaders under the wrongful assumption that these will manage to permeate the entirety of the assigned population.

*“The barrier is that the information is delivered to the council, to two, three people, and the community is our whole population of 7200.”* (HO-C5).

**2.4. Sociocultural and territorial dimensions: Aging leadership and age incompatibility.** Lastly, sociocultural determining factors emerge underpinned by the rigidity of institutional schedules and the sociodemographic profile of participants. Meetings and events are routinely scheduled in regular working hours (14:30 or 15:00, for example) that systematically exclude the young segments and those economically active.

*“Sadly younger people are working and do not have time, that’s another obstacle, maybe they want to participate, but can’t”* (HO-C5).

*“It is not that young people do not want to participate, what happens is that young people work. The meetings are at 14:30 and they are working at that hour.”* (CM-C4).

As a result, the spaces of the development council present a notorious aging of their members, composed almost exclusively of senior citizens that assume multiple community roles, resulting in a manifest phenomenon of overload and leadership burnout:

*“Leadership overloaded with meetings”* (CM-C3).

*“We lack leaders with commitment”* (CM-C4).

3. Effects and socio-institutional implications

**3.1. Quality and instrumentalization of care: “Paper thin” accreditation and loss of social belonging.** The community’s retreat from quality processes generates deep consequences in the organizational culture of the health center. The emergence of a purely procedural and instrumentalized quality is noted, focused on the standardization of technical guidelines but lacking communal legitimacy on its entirety.

*“…The main cause…is that the guidelines don’t say nothing. What we are talking about Is quality, and quality are a bunch of processes…in no part the guideline commits us or tells us to coordinate on working with the community”* (HO-C8).

*“Since there’s lower participation, we also lose the contributions that could have been made in the community…if we can help improve the quality of care…we are squandering the opportunity to get involved.”* (HO-C8/CM-C7).

This separation generates a substantial rift between “technical quality” (following institutional guidelines) and *“perceived quality”* by the user on a day-to-day basis, where basic aspects of care and biosafety are determining the real value given to the service. (*“If I am going to get checked and I see the medic who is going to treat me, and I notice they haven’t even washed their hands…”* HO-C6).

This situation generates a loss of community perspective in institutional management. The expression *“We lose the community contributions as well”* is evidence that low participation implies not only a diminished representation, but also loss of knowledge, experiences, and feedback potentially relevant to identify necessities and steer improvement processes. In parallel, the perception that *“…quality is just a bunch of processes”* and *“in no part the guidelines commit us to work with the community”* reveal a tension between accreditation logic that is centered on the achievement of standards and a view of quality that incorporates the necessities and experiences of the population as a substantial improvement component.

Lastly, the findings also show a weakening of the link and sense of belonging between FHC and community. Expressions such as *“…now all we got to do is join as a whole FHC family”*, *“…we don’t value what we have until we’re just about to lose it”* and *“my health center is one place I should value”* reveal a tension between a perceived diminished community belonging and the need to rebuild a relationship based on closeness, value and mutual responsibility. The latter is also tensioned by a perception of asymmetry in the exercise of rights and duties, expressed as *“…everyone talks about rights, but duty…that’s super difficult”*.

**3.2. Community-institution relationship: Diminished social control, apathy, and technocracy.** The scarce participation directly erodes PHC social control functions that are relevant to the citizens. At the impossibility to steer the FHC’s strategic decision making, the population shifts to a posture of apathy, skepticism, or withdrawal from public spaces:

*“…As persons we are very apathetic…very closed in within ourselves…There is a reduction in participation in social topics, not only what concerns me as a particular individual, but also me as part of an environment”* (CM-C8).

Facing this citizen disenfranchisement, a technocratic management is consolidated, where decision power is concentrated exclusively in institutional bureaucracy, feeding a delegative, passive, and paternalist attitude between the very same community leaders.

*“…Always it’s going to be determined by, let’s say, people behind the desk. So, then people get disenfranchised”.* (CM-C7).

“We as leadership fully trust those handling the FHC, we know they are very smart and responsible…” (CM-C7).

In summary, these results, paired with the effects, show that the diminished PC in the process of quality and accreditation transcend a quantitative reduction in attendance to participative instances, becoming an issue that affects social belonging to quality, community–institution links, and the community view of PHC. In this case, participants live a PC that is mainly informative, consultative, and reactive, ascribing to mechanisms such as information delivery, surveys, and complaint management while the inclusion of the community in the deliberation, decision making, and co-management of quality processes is shown to be scarcely developed.

**3.3. MCFCHC model impact.** Finally, the weakened participation generates a regression in the MCFCHC model. As the professional schedule is blocked for quality administrative talks without a community counterpart, the user perceives a restriction in medical offer that foments conflict in the waiting room. *“Blocked schedule.”* (HO-C8).

This foments a purely utilitarian, individualist relationship with FHC, far from co-responsibility in healthcare.

*“The majority sees their problems fixed and that’s that.”* (HO-C5).

In this context, community is perceived mainly as a receptor of information or as users that manifest their discomfort through the established formal channels, particularly complaints. The expression *“And when do they show up? When there are complains…”* synthesizes this predominantly reactive participation mode. In this way, social control and the extent to which the community can contribute to the evaluation, oversight, and transformation of institutional processes are diminished. Participation is also concentrated in a small group of leaders, expressed in *“…always the same leaders that work with us…”*, making the incorporation of diversity, broad voices, and experiences all the more difficult.

4. Strategies and transformative intervention proposals (Actions)

**4.1. Communicative innovation and territorial health alphabetization.** To revert this information asymmetry, the participants propose displacing institutional communication to more dynamic and visual formats beyond the workplace. Examples of this are the use of technology—for example, smart TVs showing educational capsules about quality in waiting rooms—simplifying guidelines with infographics, and the use of mass territorial media like the use of public health fairs and community “Perifoneo” (use of speakers to deliver a message).

*“Make health fairs in the public square”* (CM-C6)/*“Perifoneo through the streets”* (CM-C7), *“Write in the informative board”* (HO-C2). *“Upgrade the television, buy smart, show there the capsules.”* (HO-C5)/*“Make educational capsules”* (HO-C8)/*“Make simple infographics”* (HO-C3).

**4.2. Continuous development, bidirectional training, and reflection sessions.** In the organizational aspect, the idea of institutionalizing internal internships and permanent reflection sessions is brought up so that clinical and informative personnel understand the social dimension of quality. Likewise, training of administrative personnel in urgency categorization (SAPU/SAR) would reduce tensions as well as the opening of “Quality days” oriented towards the community.

*“We must keep internal internships and continuous reflections to socialize quality technical criteria with newer workers.”* (HO-C1, C3). *“Every year, at least a technical council, we go and give like a few presentations on quality, that we call it quality day, but towards the community”* (HO-C4).

**4.3. Co-evaluation, continuous feedback, and citizen monitoring.** To give empiric basis to participative management, we project the joint application of evaluation instruments in the waiting rooms. Examples of this are suggestion mailboxes in open view, co-designed user treatment surveys given by the same community, and performing a transparent analysis of grievances and suggestions from the technical committees and development council.

*“Survey in the waiting room while they wait for an appointment…apply the survey by the community. Construction of everything behind that survey”* (HO-C7/HO-C4). *“Put the suggestion mailbox on open view, review grievances in the committee, give account of that in the development council”* (CM-C1/CM-C6/HO-C5).

**4.4. Territorialization, reorganization of resources, and youth inclusion.** Lastly, a strategy of active territorial deployment is proposed, going further than the FHC’s walls and towards social organizations, youth organizations, and student councils to surpass the age gap. This reactivation of links with the community requires a re-structuring of assigned hours to health promotion, maximizing available resources and promoting horizontal participative diagnoses.

*“We should go right outside the FHC, and ask the people coming in three questions…give them the possibility to answer if they want to be part of development council”* (CM-C5).

*“It was proposed to accompany me to all community groups to motivate them…we should also summon…some youth association, student council, we should go with them to a consultative council.”* (HO-C1).

*“Make a participative diagnostic…where a large part of healthcare workers can participate with a large part of the community…analyze, solve, and then get a little enthusiasm.”* (HO-C4).

*“…try to work to its maximum extent…find the way of working with greater efficacy with the actual working hours and physical resources we got for prevention and promotion”* (HO-C1).

## 4. Discussion

As previously stated, this study was derived from previously obtained results from a prior study realized by Coss-Mandiola et al. [32], which motivated the implementation of this new investigation project, as a strategy that contributes to strengthening community participation in areas of quality management and accreditation. Even though the study reveals a low CM participation in activities related to the execution of the study, it cannot be stated that this low commitment and rallying towards the activities made under this project’s framework involves a low PC in other activities stemming from PHC management.

CM participation in the study is mostly female, a phenomena that can be explained by a historic appreciation of achievements both past and present that validate the work of females, strengthening its implications. This generates a social recognition that places women with greater institutional incidence, in this case, in healthcare.

As to PC being mostly composed by senior citizens with ample experience in leadership, this is part of the observed reality and only represents the perception of those who could partake in the study. Therefore, it does not represent the barriers perceived by the youth, those of working age, or other types of population that were unable to participate in the study. The attendance of the senior population can be explained, according to Martínez Rosas et al. [35], in that the integration of this population promotes healthy aging, reducing functional dependencies and strengthening support networks. Nonetheless, this valuable observed social capital evidences the challenge of diversifying the age range to secure representativity and sustainability in the process. To this end, the inclusion of new groups will be fundamental to transition from limited participation models, such as the one observed, to more inclusive and transformative ones, guaranteeing that healthcare management in this and other areas reflects the diversity of the population it tends to [36,37].

Another analysis regarding the almost exclusive PC of senior citizens is that it could constitute a mobilization or participation bias. This kind of bias is a phenomenon in which the spaces of participation end up dominated by privileged sectors, leaving out the most vulnerable population. It happens when the sample of those who attend or participate in community mechanisms is not representative of the general population but rather systemically leaning towards specific demographic and socioeconomic profiles [38,39]. In this case, in healthcare, the institutional rigidities of the rallies, such as work hours, presential formats, and technical language act as a socio-structural filter that favors the attendance of profiles with greater social capital and spare time, such as senior citizens and pensioners, invertedly demobilizing other vulnerable groups such as the youth, workers, or migrants, causing the capturing of the healthcare agenda by a homogeneous group who deprioritize the necessities of the excluded communities. It is what can be translated from the phrase *“Always the same participants”* [40].

PC is a management process viewed with a longing from what it once represented. Lastly, this PC perception also shows that the participative process somehow requires a new way of handling that allows greater efficacy. No doubt, this situation is a reality that contrasts with the Alma-Ata, which demands that healthcare systems guarantee social participation and the protagonism of organizations in healthcare decision making [41]. In other words, it can be said that the gap between declared normative and the practical execution of the PC model in healthcare remains.

Due to this motive, obtained results evidence the necessity of strengthening this model, redirecting efforts to shift from a passive PC to a more active form. In this sense, what is proposed is a double purpose objective. On one side, it is expected to improve PC levels, that is, increase the number of people that participated in PHC-related activities. But, likewise, it is also desirable to increase the degree of commitment that these activities demand from the community; in other words, it should aim to achieve greater incidence of PC in healthcare decision making. To this end, we find support in the national public policy, which notes that strengthening PC is relevant because it forms part of the *“National healthcare strategy for 2030 healthcare goals”*, adding specially to the community healthcare model and the achievement of impact goals that tend to “strengthen care focused on people, families and communities, advancing towards universal coverage” [42]. And in parallel, the strengthening of PC is appreciated as a need to offer following multidisciplinary teams to establish relationships with the community that are collaborative and horizontal, as well as the empowerment of the community to turn citizens into main healthcare and self-care agents [43].

In any case, we might be able to affirm that these PC levels coincide with what has been stated by Arnstein [8], who in her article *“A ladder of citizen participation”* describes three main blocks of participation in eight levels. In this case, PC derived from observed results correspond to the second block, which is described as a symbolic participation, a mere formality in which there are three levels: (3) information, (4) survey, and (5) conciliation. If we consider what happens to PC in topics of quality and accreditation, all CM can hear and be heard but lack the power to ensure that their observations and requirements are held in consideration by the powers that be, in this case, the entire healthcare system machinery that moves the healthcare quality accreditation system, both national and local.

This is evident as we appreciate the participants’ discourse; there is still a gap in the orientations by the health ministry and the accreditation standard guidelines which prioritize technical and clinic metrics, omitting PC as a mandatory requirement. In this regard, the only standards that some metrics include (in technical trait terms) are those regarding patients’ dignity and safety in the installations, in which we highlight aspects relating to the management of complains and participation on emergency drills as the only ones that narrowly include PC [41]. This situation makes it so that healthcare teams perceive accreditation as a technical-clinical task foreign to the user. All this allows us to notice the prevalence of a paternalist model where HOs assume the community is a passive entity and mere receiver of healthcare. This vision is also shared by CMs who perceive accreditation as an almost exclusive process of healthcare teams, leaving their roles as community agents to a strictly reactive or secondary one. This situation can be explained by a recent Spanish study that found that the valuation of those surveyed about improvement alternatives to the healthcare system are aimed to a deficit in institutional participation. That is, those who use healthcare resources consider that the responsibility of the system’s progress is held fundamentally by medical personnel and not politicians, public administration or consumer/user groups [6].

Furthermore, a gap in PC rallying perception has been evidenced as the community perceives minimal and insufficient representation in spaces that they should be present. Likewise, some HOs attribute the lack of attendance to a community “Mea Culpa” derived from lack of interest and commitment of registered users. They too manage to identify certain moments where this community exclusion turns explicit, such as commemorative accreditation events where some community leaders complained to not have been involved in prior work. These highlights have allowed healthcare teams to recognize PC as an unsolved necessity and a priority gap to be bridged. Though, if this perception was to be improved, perhaps it is worth adopting the views of Abramovich [44], in relation that PC should be framed in what it is denominated “focus on rights in politics and development strategies” and from this view, it must be procured to change the logic of the policy-creating processes so that the starting point is not the existence of people with necessities that must be satisfied, but subjects with the right to require determined actions, services, and conducts.

In this regard, the concept of a *“Critical awakening”* appears, in this case from the HOs, caused by the *“awakening”* of community members in the form of an uproar as they were not considered in the accreditation process. This concept is based on the work of Paulo Freire, in which he promotes that there must always be a constant, educative, effective, and critical dialogue which leads to thinking about reality and making efforts for it’s transformation [45]. This practice is centered around popular education, which aims to favor coexistence with the dynamics of healing and disease within the community, the interaction with local social movements and the participation in activism of other intellectual groups to achieve a re-orientation of care practices, seeking to handle health issues in a more holistic manner [46]. Therefore, the phenomena being present in both CMs and HOs, can be appreciated as promising to favor PC in topics of quality and accreditation.

This perception of the issue can be an indicator that both HOs and CMs can perceive the need for a change in methodology for handling the PC process in general and specific topics of quality management and accreditation. These results can be revealing a gap in the normative framework of MCFCHC and the reality of FHCs, where PC, even though declared as the central axis of local management, is limited to consultative and informative levels without reaching citizens’ empowerment [9,16,18].

In this way, social control as well as the community’s capacity to systematically contribute to the evaluation, oversight and transformation of institutional processes are weakened. Social control is understood as the right and duty of citizens who, as an exercise of their ability to participate, seek binding with state power with the end of controlling the proper handling of its administration, with the ultimate goal of having a say in public policy decision making at all management levels [47]. In this case, for subjects regarding quality management and accreditation, PC remains a non-binding space in decision making, diminishing empowerment and social control in healthcare [48].

Another decisive factor is a lack of explicit demands in the current standards to include community leaders as key actors. Notwithstanding, adjustments are promoted to incorporate jointly built community participation metrics, facilitating integrated care and centered in the individual [49]. It is vital that these standards are differentiated by care level, thus securing that they are pertinent, verifiable, and applicable to different specific operational realities of both open and closed attention.

Still, the discourse allows recognition that low participation is not exclusively due to community disinterest, as there is also the intervention of institutional, communicative, generational, and material barriers that condition the real possibilities of participation.

The stated can be supported by a Spanish study that analyzed user complains in healthcare management, which contributes a double outlook. On one side, regarding grievance management, the study points out that complaints receive a purely bureaucratic answer, that is, no investigation took place to know the causes and context of the incident, limiting themselves to an apology and/or change in the professional involved [50]. The first interpretation suggests that this can be a factor that reduces community participation through a lower complain rate. On the other side, the study concludes that work should be done to prioritize the notification of complaints and grievances from the patients, as well as improvement suggestions that the healthcare team can make, as that leads to safety improvements in healthcare.

To point that out is to state that these multiple barriers observed in the PC process invite a change in the way the process is view, a change that favors relevant transformation of this PHC management process. If this change does not take place, we will possibly be quite far from what is observed in countries like Norway, England, the Netherlands, and Australia, where PC in quality and safety is not limited to reactive participation methods such as leaving complains, but the incorporation of proactive individual and collective PC methods in subjects like regulations [51].

On another note, results place these phenomena in a context of tension within the Primary Care Model, participants perceive that FHC *“…is now solely for treatment and rehabilitation…”* and that it had *“lost the point of what PHC was.”*, identifying a shift in promotion, prevention, and community work towards a growing centralization of care. Thus, the diminishing participation and the weakening of the community component in the model appear as related phenomena that can mutually reinforce each other. The results reinforce that participation by healthcare teams is the center of the process, while community participation is limited or symbolic [32]. The current normative framework restricts the user’s role to the management of grievances and safety, without systematically integrating them into the evaluation and improvement of the healthcare management processes [21].

International, it is warned that the lack of methodological coherence and institutionalization of the PC in healthcare generates uncertainty about its reach and sustainability, directly affecting the impact in healthcare management [3,7]. According to evidence, this lack favors the misuse of community resources and negatively conditions the acceptance of health policies, diminishing the sense of belonging and compromising the sustainability of healthcare and accreditation improvements.

On another note, the PAR methodology facilitated horizontal dialogue and collective construction, proving its transformative potential in complex institutional contexts, as is the healthcare area [52]. Furthermore, international evidence shows that community participation integrated in healthcare management improves accountability, perception of quality, and patient safety [2,4]. This collaborative focus on care evaluation promotes user trust in providers, which is in itself a fundamental cornerstone to foster PC in healthcare programs [7]. Initial results confirm that PC in quality and accreditation of PHC is a key challenge in public health and a fertile ground for innovation. In this line, the study also shows encouraging proposals that contain several dimensions; information, formation, training, management, decision making, organizational strengthening, and financing, among others, in agreement with what has been stated by Wiig et al. [51]. To achieve these goals is complex, but decision makers can experiment, innovate, and evaluate diverse participation methods as these actions will allow them to extract invaluable lessons to continuously optimize community involvement in healthcare, much more so regarding quality and healthcare accreditation.

The study also evidenced the need to strengthen conversational and leadership skills in both healthcare teams and the community, thus justifying the addition of a leadership course based on strategies derived from ontological coaching. On this, diverse research supports the direct impact of this training in healthcare management, in which the development of leadership, teamwork, communication, and strengthening of nurses’ voices as well as multidisciplinary collaboration are key aspects that facilitate the improvement of PHC and the success of local accreditation [53,54]. In turn, handling this training from an ontological perspective, according to Echeverría [55] provides a space of deep reflection that allows the shift from experience to concept, the exercise of pondering and comprehending the phenomenon in depth; PC, in this case, allows the design of effective strategies that allows improving the PC model in quality management and accreditation.

Finally, it can be raised that the use of PAR and the logic frame method allowed the systematic organization of issues, causes, and effects, as well as drafting proposals for action coherent with quality standards and MCFCHC principles. In this sense, the study provides empiric evidence that supports the need to advance towards accreditation models that incorporate the community voices in an explicit, binding manner, not just as end users, but also as co-responsible key actors of the healthcare system.

### Study Limitations

The main limitations of the study are linked to its qualitative design-bases PAR investigation, which restricts the generalization of results to all FHCs in the country. Distinctly, the community sample focused primarily on senior leaders which omitted perspectives from other age ranges. In this case we face a known, recognized representativity limitation as PC was mostly composed of LDC leaders with an average age of 67.9 (and 69.4% over the age of 65), in its majority retired/pensioners. It is recognized that this composition is product of channeling the invitation through municipal healthcare authorities and existing leadership structures, as this may have introduced a selection bias towards people with greater prior institutional implication, sub-representing the youth, the working aged and other community groups such as non-committed immigrants. Likewise, the high care load of healthcare workers made a more diverse participation in the technical team difficult. These conditions suggest the findings reflect primarily the experiences and proposals of those who were able and available to participate.

Another limitation can be related to the inclusion of just eight accredited FHCs. In that regard, it is clarified that the project originates from accredited FHCs due to one of the traits of these health centers being their location within municipalities of low socioeconomic level compared to others and, even so, earned their accreditation. This being the reason the study was centered around them, with the purpose of determining which aspects allowed these centers to have successful accreditations. The next purpose is to share their experience with other unaccredited FHCs. A different limitation can be the territorial restriction to just two municipalities in two regions. This is due to these municipalities having accredited as well as having limited resources. For this reason, it was fundamental to continue developing work based on an issue previously identified in these same locations by healthcare teams.

A further limitation implies a potential social desirability bias in the qualitative interventions. To address it and avoid this bias, mediators employed questions not logically connected to one another to avoid making the participants feel evaluated themselves. To complement this, the researchers declared in more than one opportunity that they were not evaluating the participation process of healthcare teams nor the community.

A possible limitation related to the impact the prior 4-year-old relationship of collaboration between the and the research team. To address this, it is worth highlighting that research work in and their communities may be difficult, thus, having antecedents from prior investigations with these healthcare teams favored access to the and their communities to implement this participatory action research-based investigation. The experience in focal groups evidenced that the community did not limit their answers or omit information regarding the difficulties they have articulating between them and the healthcare teams; the impact analysis will be developed in later stages.

Lastly, in relation to the actions proposed as improvement strategies that were able to be obtained from the study, it is made clear that they constitute part of the diagnostic and their analysis is only limited to their classification, and as such, the impact analysis will be developed in later stages.

## 5. Conclusions

The investigation allows us to conclude that participation of the community in PHC quality management and accreditation is still incipient, scarce, and insufficient to reach influential levels in decision making. There is a persistant gap between the normative framework and the real work of healthcare teams with their communities. Despite international mandates such as the Alma-Ata declaration and national frameworks such as the MCFCHC which establish PC as an irrevocable strategic cornerstone in PHC by its acronym in Spanish, the study gathered evidence in the eight accredited FHCs of Copiapó and Chillán that revealed a profound dissonance between normative discourse and day-to-day praxis. When evaluating the degree of PC involved, extracted from the participants’ voices and filtered by Arnstein [8] community participation ladder, the research shows that PC in topics of quality and accreditation is situated squarely in the block corresponding to symbolic participation or appeasement. In this sense, current PC mechanisms in these matters are reduced to initial levels of information, passive survey, and formal conciliation.

In healthcare practice, the community is summoned only reactively to answer satisfaction surveys or channel dissatisfaction through the Office of Information, Grievances, and Suggestions, lacking deliberative power or true capability to co-design oversight or evaluate quality standards and healthcare accreditation.

The study also evidences that low PC level is not a fortuitous fact, nor attributable to just mere civic disinterest, but a direct outcome of an interconnected repertoire of systemic barriers. Highlighted among them aspects of various subjects, some related to healthcare teams, others related to the community, such as: rigidity in accreditation guidelines, overload for care workers, productivism/paternalist logic, incompatible schedules, generational gap, technical hermetism regarding the accreditation process, deficient communication, and lack of various kinds of resources.

This observed low PC perception can have diverse effects or consequences, such as: diminishing the sense of belonging to the community, lack of social pertinence of quality and accreditation, weakening of MCFCHC, and consequently a regression of the PHC model to a mending logic, conflicting channeling of user dissatisfaction, and weakening of oversight and social control. This last one can further complicate the issue as it facilitates the lack of co-management spaces and reduces oversight or community control, increasing mistrust of users towards their FHC, generating a perception of decision making “Behind closed doors”.

Nevertheless, results also show that FHC healthcare teams in the study, as well as community leaders, have both accumulated relevant experience regarding participative work, defined by an important and fundamental degree of commitment and contribution which offers a very favorable base to potentiate the implementation of a model that strengthens PC in quality and accreditation for PHC. All of this is evidenced in important action proposals for improvements born out of the voices of the participants, both HO and CM.

In virtue of the stated, FHCs quality management and accreditation cannot be understood as merely a self-referent homologation of clinical and administrative procedures. If quality processes and accreditation do not actively integrate the perspective, experience, and oversight of the community it serves, then it runs the risk of consolidating a technically accredited but socially distant system. Therefore, to align with successful international experiences (where the community participates in healthcare regulation) and to fulfill the healthcare goals set for 2030 by the national healthcare strategy, PHC in Chile must shift decisively from passive, reactive participation towards a substantive co-management model, placing the citizens as bearers of rights and duties, jointly responsible in the construction of their own health.

## Figures and Tables

**Table 1:** Participants’ traits.

Region	Municipality	FHC	HO			CM			Total
			**Women**	**Male**	**Subtotal**	**Women**	**Male**	**Subtotal**	
Atacama	Copiapó	Candelaria de Rosario	14	3	17	3		3	20
		Paipote	8	2	10	2	1	3	13
		Juan Martínez	6		6	5	5	10	16
		Pedro León Gallo	3	5	8	3	1	4	12
Ñuble	Chillán	Isabel Riquelme	5	3	8	6	2	8	16
		Ultraestación	2	1	3	4		4	7
		Los Volcanes	5	2	7	3		3	10
		Sol de Oriente	6	5	11	1		1	12
Total			49	21	70	27	9	36	106

**Table 2:** Summary of emerging topic architecture, articulating analytic categories, sub-categories, in vivo codes, and the interpretative lectures.

Main Category	Analytic Sub-Category	Emerging In Vivo Codes	Summary and Qualitative Interpretation
1. PC Perception	1.1 PC in general PHC activities.	“Participation is scarce”, “It is very hard to rally the people”, “It is always the same leadership”, “They do not want that participation all that much”, “Too limited, too partial”, “They resist to attend”, “Promise too much, deliver little”.	Broad diagnosis of fragility and rally bias. Traditional leadership worn down as institutional unkept promises have derived in citizens feeling disenfranchised.
	1.2 PC in quality management and accreditation.	“I see little attendance”, “Not so active”, “Should involve more people”, “Haven’t been active/Mea Culpa”, “A passive entity”, “I had an idea/Not even included”, “A perceived gap”, “Still a long way”, “The Community doesn’t intervene/Nothing to do”	Twin participation problem: Historic invisibility of community (considered a passive, receptive entity) and the eventual revelation of a “felt gap” of excluded citizenry.
2. Causes and determining factors for Low PC	2.1 Institutional, normative, and financial dimension.	“Quality guidelines do not require community participation”, “I wasn’t oriented”, “We focus on clinical aspects”, “We do not have the time resource”, “We do not have physical space”, “Participation is but a lie without budget”, “We lack a protected schedule”, “It is hard to reach the community”, “They have reduced our resources/We are seen as numbers.”	Explicit omission of PC in normative instruments of quality accreditation. Dominance of biomedical logic and asphyxiation of community time to achieve quantitative performance goals (primary care performance metrics or Primary Health Care Activity Indicators (PHCAI)).
	2.2 Information management and communications dimension.	“Lack of knowledge”, “People do not get it/do not fully get it”, “We do not get the information”, “Lack of digital means”, “We reach 2, 3 people.”	Information asymmetry and hermetism of the technical lexicon of the accreditation. Diffusion channels are highly concentrated and ineffective to mobilize the general user base.
	2.3 Territorial and sociocultural dimension.	“Younger people are working”. “Meetings are at 14:30”, “Low interest”, “Overloaded leadership”, “Lack of commitment in leadership”.	Rigid institutional work schedules that exclude the working/studying age youth, causing an aging, overloaded leadership.
3. Effects and socio-institutional implications	3.1 Quality and service instrumentalization.	“Diminishing social issues”, “There is no way to be involved with the script”, “I don’t see how it can work”, “Too few results”, “Missing the opportunity”, “Quality are just a bunch of processes”, “We squander the contributions”.	Decoupling between standardized technical quality and social pertinence. Risk of “paper thin” formal accreditations that lack citizens’ co-construction.
	3.2 Community–institution link.	“Absolute lack of knowledge” “We are quite apathetic” “Those people behind the desk”, “Trust them fully”.	An erosion of social control in healthcare. Apathy and lack of understanding coexist with paternalistic delegation and disenfranchisement from technocratic decisions.
	3.3 MCFCHC model impact.	“Blocked schedule”, “Solves the problem and that’s it.”	Devolves towards a reductionist immediate demand focused on contingent medical issues, debilitating the focus on pre-emptive, integral medicine.
4. Courses of action and strategic proposals	4.1 Communication, health alphabetization and diffusion.	“Health fairs”, “Perifoneo”, “Educational capsules/use of smart TVs”, “Simpler infographics”, “Informative board”.	Diversificación de medios Diversify territorial media and pedagogically translate accreditation for the regular user through ludic digital tools.
	4.2 Governance, capacitation, and participative monitoring.	“Internal internships/Continuous reflection”, “SAPU/SAR courses”, “Day geared towards quality to the community”, “Suggestion Analysis”, “Evaluate rights”, “Waiting room surveys”, “Visible Mailbox”, “User treatment surveys”, “Committee complain reviews”.	Technical empowerment of the user through quality days and co-administered surveys in waiting rooms. Institutionalized continuous feedback.
	4.3 Youth inclusion, territorialization and re-orientation of resources.	“Insist on promoting outside of FHC”, “Rally youth groups”, “Joint group discussions”, “Rekindle workmates/leaders”, “Work with greater efficiency/efficacy”, “Participative diagnostic”	Deploy beyond the walls of FHC, reactivate social fabric in youth and integrate participative diagnostics to guide efficient management of resources assigned to promoting.

## Data Availability

The data will be made available in an anonymized format, upon request to the corresponding author, in order to maintain the confidentiality of the information, as required by the Institutional Ethics Committee.

## Abbreviations

The following abbreviations are used in this manuscript:

CM	Community Members
COREQ	Consolidated criteria for Reporting Qualitative research
FHC	Family Health Center
FHCs	Family Health Centers
HO	Health Officials
LDC	Local Development Councils
MCFCHC	Model of Comprehensive Family and Community Health Care
PAR	Participatory Action Research
PC	Participation by the Community
PHC	Primary Healthcare
PHCAI	Primary Health Care Activity Indicators
WHO	World Health Organization
